# Renal transplant patients with preformed anti-HLA antibodies: early biopsy findings and clinical outcomes^a^

**DOI:** 10.1590/2175-8239-JBN-2018-0244

**Published:** 2019-09-12

**Authors:** Marcos Vinicius de Sousa, Ricardo de Lima Zollner, Marilda Mazzali

**Affiliations:** 1 Universidade de Campinas, Departamento de Clínica Médica, Faculdade de Ciências Médicas, Laboratório de Investigação em Transplante, Campinas, SP, Brasil.; 2 Universidade de Campinas, Faculdade de Ciências Médicas, Departamento de Clínica Médica, Laboratório de Imunologia Translacional, Campinas, SP, Brasil.

**Keywords:** HLA Antigens, Graft Rejection, Fibrosis, Proteinuria, Reperfusion Injury, Biopsy, Antígenos HLA, Rejeição de Enxerto, Fibrose, Proteinúria, Traumatismo por Reperfusão, Biópsia

## Abstract

**Introduction:**

Renal fibrosis is the end point of a process that begins at transplant, with ischemia reperfusion and early inflammation, and progresses over time with immunological and non-immunological phenomena. Early identification of morphological markers and intervention could improve graft function and survival.

**Objective:**

to evaluate the correlation between intensity and specificity of pre-transplant anti-HLA antibodies and kidney allograft pathology in order to identify early risk factors or markers of allograft dysfunction.

**Methods:**

A retrospective cohort of kidney transplant recipients with pre-transplant anti-HLA antibodies who underwent graft biopsy within the first two years post-transplant was divided into two groups according to the specificity of anti-HLA antibodies: nonspecific (non-DSA, n = 29) and specific (DSA+, n = 16). Kidney graft pathology, renal function, and proteinuria were analyzed.

**Results:**

general characteristics were similar in both groups, except for the higher dose of thymoglobulin in DSA+ group (*p* < 0.05). The non-DSA group had higher scores for glomerulosclerosis, interstitial inflammation (i) and interstitial fibrosis (ci) (*p* < 0.05) and higher incidence of cell-mediated acute rejection. No statistical difference in incidence of antibody-mediated rejection, renal function, and proteinuria was observed during follow up.

**Discussion and conclusions:**

the difference in inflammation scores and interstitial fibrosis may be associated to the higher incidence of acute cell-mediated rejection and polyomavirus nephropathy in the Non-DSA group. We also should take into account the protective effect of higher doses of thymoglobulin, reducing ischemia reperfusion injury in the DSA+ group. The short follow-up might have been insufficient to detect long-term changes in allograft tissue, renal function, and proteinuria.

## Resumo

**Introdução:**

A fibrose renal é o desfecho de um processo iniciado no transplante, com reperfusão, isquemia e inflamação precoce, que progride ao longo do tempo com fenômenos imunológicos e não imunológicos. A identificação de marcadores morfológicos e a intervenção precoce poderiam melhorar a função e a sobrevida do enxerto.

**Objetivo:**

Avaliar a correlação entre intensidade e especificidade de anticorpos anti-HLA pré-transplante alterações histológicas do enxerto renal, de forma a identificar fatores de risco ou marcadores de disfunção precoces do aloenxerto.

**Métodos:**

O presente estudo incluiu uma coorte retrospectiva de receptores de transplante renal sensibilizados com anticorpos anti-HLA no pré-transplante submetidos a biópsia de enxerto nos primeiros dois anos após o transplante. Os grupos foram divididos em função da especificidade dos anticorpos anti-HLA: sem anticorpos doador-específicos (não-DSA, n = 29) e com anticorpos doador-específicos (DSA+, n = 16). Alterações histológicas do enxerto renal, função renal e proteinúria foram analisados.

**Resultados:**

Os dois grupos tinham características gerais semelhantes, exceto pela dose mais elevada de timoglobulina administrada nos indivíduos do grupo DSA+ (*p* < 0,05). O grupo não-DSA teve escores mais elevados de glomeruloesclerose, inflamação intersticial (i) e fibrose intersticial (ci) (*p* < 0,05), além de maior incidência de rejeição celular aguda (RCA). Não foi observada diferença estatística na incidência de rejeição mediada por anticorpos, função renal ou proteinúria durante o seguimento.

**Discussão e Conclusões:**

A diferença nos escores de inflamação e fibrose intersticial pode estar associada à maior incidência de RCA e nefropatia por poliomavírus no grupo não-DSA. Devemos considerar ainda o efeito protetor das doses mais elevadas de timoglobulina na redução da lesão por isquemia-reperfusão no grupo DSA+. O curto período de seguimento pode ter sido insuficiente para detectar alterações de longo prazo no tecido do aloenxerto, função renal e proteinúria.

## Introduction

Renal fibrosis is considered the common end point of countless disease processes in renal transplantation, resulting in progression of kidney disease and graft loss^1^^,^ 2. Potential causes of fibrosis include drug-induced nephrotoxicity, ischemic injury, and immune-mediated lesions. The fibrosis process begins throughout the first year post-transplant, especially within the first three months, triggered by self-limiting inflammation secondary to reperfusion injury and maintained by continuous inflammatory immune response that facilitates progression of renal disease^2^^,^ 3 Several factors are involved in the intensity and extent of fibrosis, such as donor age, donor source (living vs. deceased), and cold and warm ischemia times. Increased donor age is considered a key predictor of worse graft outcome^2^.

After reperfusion, under the influence of proinflammatory cytokines, several cell types, including macrophages, T cells, and tubular epithelial cells, produce profibrotic mediators, such as TGF-beta, resulting in irreversible tubular atrophy (TA), excessive interstitial fibrosis (IF), microvascular rarefaction, and glomerulosclerosis^2^. In the Banff classification of renal allograft biopsies, chronic lesions include interstitial fibrosis (ci), tubular atrophy (ct), arterial fibrous intimal thickening (cv), arteriolar hyalinosis (ah), increase of mesangial matrix (mm), and transplant glomerulopathy (cg)^2^. A higher score of IF/TA in the graft biopsy is associated with worse renal function, with a negative impact on graft survival.

Immune sensitization, defined by the presence of anti-human leukocyte antigen (HLA) antibodies in the recipient’s blood, is triggered by prior exposure to HLA antigens, usually through previous organ transplant, pregnancy, or blood transfusion^4^. Sensitized recipients have an increased risk for antibody-mediated rejection (AMR) after renal transplantation^1^. Biopsy-proven acute rejection is considered a risk factor for IF/TA^2^. Also, subclinical AMR, suggested by the presence of donor-specific antibodies (DSA) and histological findings of glomerulitis, peritubular capilaritis and/or C4d deposition, without graft dysfunction, is a powerful profibrotic stimuli and can predict graft loss^2^. Even though T cells are usually the most abundant infiltrating cells, several other mononuclear cells, such as B cells, natural killer cells, dendritic cells, and monocytes/macrophages contribute to the pathological process. There is evidence of a worse prognosis with the presence of natural killer cells (strongly associated with AMR), dendritic cells, and macrophages (increased in more severe acute rejection and associated with tubular dysfunction and chronic histological damage)^2^. Therapeutic strategies to minimize progression of fibrosis in renal graft include: calcineurin inhibitors avoiding or minimization regimens, blockade of the renin-angiotensin-aldosterone system, and treatment of subclinical rejections^2^. The incidence of clinical rejection in the first year post-transplantation was reduced with modern immunosuppression^3^, and the diagnosis of subclinical rejections and identification of early markers of fibrosis became important predictors of renal function and graft survival.

The aim of this study was to evaluate the correlation between intensity and specificity of preformed anti-HLA antibodies (classes I and II) and renal pathology in graft biopsies performed within the first two years after renal transplantation, in order to identify risk factors potentially associated with the onset of graft fibrosis and early clinical outcomes.

## Materials and methods

### Patients and inclusion criteria

This was a single center retrospective cohort of renal transplant recipients with preformed anti-HLA antibodies.

Inclusion criteria: renal transplant recipients of living or deceased donors, older than 18 years old at the time of transplant; presence of pre-transplant anti-HLA antibodies (classes I and II) and graft biopsy within the first two years after transplant. Exclusion criteria: pediatric recipients, absence of anti-HLA antibodies before transplantation (PRA=zero), or not having a graft biopsy within the first two years after transplantation.

All selected renal transplant recipients had a negative pre-transplant crossmatch by complement-cytotoxicity (CDC). Crossmatch by flow cytometry was not performed. Recipients from standard and expanded criteria donors were included, according to the criteria proposed by the United Network for Organ Sharing (UNOS) in 2003^5^.

End points were graft biopsy morphology, renal function, and presence of proteinuria within the first 2 years post-transplantation. The study population was divided into 2 groups according to the presence of anti-HLA antibodies before transplant: DSA+, with donor specific antibodies; and Non-DSA, with non-specific anti HLA antibodies. The study protocol was approved by University of Campinas Ethics Committee (CAAE: 51485415.6.0000.5404).

### hla typing and detection of hla-antibodies

Receptor and donor HLA were typed by deoxyribonucleic acid (DNA) amplification by Polymerase Chain Reaction (PCR) in peripheral blood samples with molecular primers sequences (LABType™ SSO and Micro SSP™, One Lambda Inc, California - USA) following manufacturer’s instructions. The generated file was imported into the HLA fusion software (One Lambda Inc, California - USA) for analysis. HLA-A, -B, and -DRB1 were routinely identified and, in cases where recipients also had anti-DQ antibodies, donor HLA-DQ antigens were also identified.

For detection of anti-HLA antibodies, recipient’s peripheral blood samples collected before and after transplant were incubated with microspheres labelled with classes I and II HLA antigens (LABScreen™ Single Antigen HLA Class I LS1A04 and LABScreen™Single Antigen HLA Class II LS2A01). Panel-reactive antibody (PRA) was calculated based on the prevalence of HLA alleles of organ donors from Sao Paulo - Brazil, which is usually updated every six months and had about 2,750 records by the time of the study.

Anti-human globulin-enhanced complement dependent cytotoxicity negative T-cell crossmatches and NIH complement-dependent cytotoxicity B-cell crossmatches were required for all kidney transplant recipients at the time of transplant, performed with the most recently collected serum and donor cells obtained from lymph nodes or spleen. In case of recent recipient’s vaccination or transfusion, an additional CDC test was performed with another serum sample collected at the time of convocation for transplant. Only patients with negative CDC for T and B cells received a kidney transplant. All donor-recipient pairs were ABO-compatible.

According to service guidelines and regional and national laws, all kidney transplant recipients were typed and screened for anti-HLA antibodies at the time of entering the transplant waiting list. The PRA was calculated every six months and the transplant candidate serum was collected every three months for possible crossmatch testing. Information obtained by Luminex™ was used to guide post-transplant immunosuppressive strategies and define the need for monitoring these antibodies during follow-up.

### Immunosuppressive therapy and diagnosis of rejection

Induction immunosuppressive therapy for recipients from standard kidney donors and with low immunologic risk consisted of monoclonal anti-IL-2 receptor antibodies and 20 mg Basiliximab IV, on the day of transplantation and on the fourth day post-transplant. For recipients of kidneys from expanded criteria donors, non-identical HLA living donors, or considered of high immunological risk (PRA > 50% or presence of DSA), induction therapy was 3 to 7 mg/kg anti-thymocyte globulin IV adjusted for total lymphocytes count. All recipients received 500 mg methylprednisolone IV at the time of transplant and remained on steroid therapy during the follow-up. Maintenance immunosuppression consisted of a combination of calcineurin inhibitor (0.1 mg/kg tacrolimus bid, dose adjusted according to blood levels) and 720 mg sodium mycophenolate bid, adjusted according to body surface, gastrointestinal tolerance, and white and red cell count in peripheral blood. None of the included patients received desensitization protocol before transplantation.

Rejection was suspected by increase in serum creatinine (> 20% from baseline level) or new onset proteinuria, and confirmed by allograft biopsy, according to Banff 2013 classification^6^. Renal biopsies were tested for deposits of C4d in peritubular capillaries by peroxidase staining protocol^7^. Diagnosis of antibody mediated acute rejection (AMR) was based on histologic criteria and presence of donor-specific antibodies. Biopsy reports were obtained from medical records and were not reviewed for the study. Graft biopsies are routinely evaluated by two experienced renal pathologists.

### Collection of clinical data and outcomes

Clinical and laboratory data were retrospectively collected from medical records and Renal Transplant Program databases at the time of transplantation and at 1^st^, 3^rd^, 6^th^, 12^th^, 18^th^, and 24^th^ months after transplant. Data was recorded and organized into a Microsoft™ Excel worksheet. Primary outcomes were graft biopsy characteristics, according to Banff 2013 classification 2015 revision^8^, allograft function, estimated using the study equation of the Chronic Kidney Disease Epidemiology Collaboration (CKD-EPI)^9^1999 to 2006. PARTICIPANTS: 8254 participants in 10 studies (equation development data set, and proteinuria, expressed by protein/creatine ratio in urine sample.

### Statistical analysis

Numerical data is reported as the mean + standard deviations, median and range, and/or percentages. Continuous variables among the groups were compared using Kruskal-Wallis test, whereas categorical variables were compared using Pearson x^2^ tests. Values of *p* < 0.05 were considered statistically significant. Data were analyzed with the GraphPad Prism 7.0c™ for Mac (La Jolla CA, USA).

## Results

### General characteristics according to anti-hla antibodies before transplantation

From 89 patients with preformed anti HLA antibodies, forty-five (50%) fulfilled the inclusion criteria. Forty-four patients without graft biopsy within 2 years after transplant were not included. Compared to the study group, non-included patients had a higher rate of living kidney transplants, less kidneys from expanded criteria donors and reduced incidence of delayed graft function. We also noticed a lower percentage of patients receiving mycophenolate. All other analyzed parameters were comparable between groups (Table 1).

**Table 1:** General characteristics of groups according to the presence of anti-HLA antibodies before transplantation

	Total non- included (n = 44)	Total included (n = 45)	*p*	Included Non- DSA (n = 29)	Included DSA+ (n = 16)	*p*
*Transplant recipientes*						
Age (years)	45.7 ± 10.4	45.1 ± 11.6	0.79	47.0 ± 12.6	41.6 ± 9.0	0.14
Male, n (%)	13 (29.5)	22 (48.9)	0.06	13 (44.8)	9 (56.2)	0.97
Etiology of CKD (%)			0.91			0.99
Unknown	16 (36.4)	13 (28.9)		9 (31.0)	5 (31.2)	
Systemic arterial hypertension	7 (15.9)	7 (15.6)		5 (17.2)	2 (12.5)	
Chronic glomerulonephritis	8 (18.2)	5 (11.1)		2 (6.9)	3 (18.7)	
Diabetes mellitus	4 (9.1)	2 (4.4)		2 (6.9)	0 (0)	
Others	9 (20.4)	18 (40.0)		11 (37.9)	6 (37.5)	
Length of dialysis (months),	47.2 ± 42.4	50.4 ± 40.2	0.71	53.8 ± 44.6	43.9 ± 30.1	0.66
Transfusions pre- transplant, n (%)	21 (47.7)	25 (55.5)	0.54	14 (48.3)	11 (68.7)	0.78
Previous transplantation, n (%)	3 (6.8)	8 (17.8)	0.11	3 (10.3)	5 (31.2)	0.54
Women with pre- transplant pregnancies, n (%)	19 (79.2)	18 (78.2)	0.18	13 (81.2)	5 (71.4)	0.99
HLA ABDR Mismatches	3.2 ± 1.5	3.3 ± 0.8	0.69	3.2 ± 0.9	3.4 ± 0.7	0.65
Pre-transplant Class I PRA (%)	42.6 ± 32.0	32.7 ± 28.9	0.13	31.4 ± 26.7	35.1 ± 33.2	0.94
Pre-transplant Class II PRA (%)	24.0 ± 32.3	28.3 ± 35.3	0.55	19.1 ± 29.4	45.1 ± 39.8	0.02
*Donors*						
Deceased donors, n (%)	32 (72.7)	41 (91.1)	0.02	28 (96.5)	13 (81.2)	0.56
Age (years)	39.1 ± 14.4	46.6 ± 12.6	0.01	46.6 ± 13.3	46.7 ± 11.8	0.97
Male, n (%)	25 (56.8)	24 (53.3)	0.74	17 (58.6)	7 (43.7)	0.92
Expanded criteria donors (%)	8 (18.1)	23 (51.1)	< 0.01	17 (58.6)	6 (37.5)	0.76
Serum creatinine (mg/dL)	1.1 ± 0.7	1.4 ± 1.0	0.10	1.4 ± 1.1	1.3 ± 0.9	0.53
KDPI index (%)	56.9 ± 28.3	58.0 ± 28.5	0.85	59.4 ± 26.3	57.4 ± 29.8	0.83
*Transplantation*						
Initial immunosuppressive therapy						
Thymoglobulin (%)	31 (70.4)	36 (80.0)	0.29	22 (75.8)	14 (87.5)	0.93
Thymoglobulin dose (mg/kg)	4.9 ± 2.2	4.5 ± 2.5	0.42	5.2 ± 1.1	6.2 ± 1.1	0.01
Basiliximab (%)	5 (11.4)	9 (20.0)	0.26	7 (24.1)	2 (12.5)	0.03
Tacrolimus (%)	36 (81.8)	40 (88.9)	0.34	26 (89.6)	14 (87.5)	0.99
Mycophenolate (%)	33 (75.0)	44 (97.8)	< 0.01	29 (100.0)	15 (93.7)	0.76
Cold ischemia (hours)	19.5 ± 4.1	22.4 ± 6.2	0.01	22.5 ± 6.8	22.1 ± 4.9	0.86
DGF, n (%)	10 (22.7)	31 (68.9)	< 0.01	20 (68.9)	11 (68.7)	0.99

n, number; CKD, chronic kidney disease; HLA, human leukocyte antigen; PRA, panel-reactive antibody; KDPI, kidney donor profile index; DGF, delayed graft function.

Considering the specificity of pre-transplant anti-HLA antibodies, twenty-seven patients presented DSA and sixty-two patients had non-DSA antibodies. Eleven patients (40%) in the DSA group and thirty-three patients (53%) in Non-DSA group were excluded because they had no graft biopsy performed (*p* = 0.28). Forty-five renal transplant recipients fulfilled the inclusion criteria: DSA+ group (n = 16) and Non-DSA group (n = 29).

The majority of patients were male, and groups were comparable for age, etiology of chronic kidney disease, length of dialysis, number of previous sensitizing events (transfusion, previous transplantation, or pregnancies). The number of A, B, and DR mismatches was similar in both groups, as well as for class I PRA. However, class II PRA was statistically higher in the DSA group (*p* < 0.05). As expected, according to our protocol, the majority of patients in both groups received thymoglobulin for induction of immunosuppression, with higher doses in DSA+ group (*p* < 0.05). Cold ischemia time and donor characteristics were comparable between groups, as well as the incidence of delayed graft function (DGF) Table 1.

### Histological features of post-transplantation graft biopsies according to groups

Seven recipients from the Non-DSA group (24.1%) and eight (50.0%) from the DSA+ group underwent more than one graft biopsy during follow-up, and all of them were considered for analysis, which resulted in 38 graft biopsies analyzed in the Non-DSA group and 29 in the DSA+ group. Graft biopsies were indicated by development of *de novo* DSA or increase in the intensity of fluorescence (MFI) of preformed antibodies (n = 3), delayed graft function (n = 29), graft dysfunction (n = 26), proteinuria (n = 3), and/or control after acute rejection treatment (n = 3), without statistical difference between groups. Despite the absence of a routine for protocol biopsy in our center, three biopsies in the DSA group were surveillance biopsies before hospital discharge. The mean overall time of biopsy was similar for both groups.

In the general analysis of graft biopsies, DSA+ group presented lower interstitial inflammation compared to Non-DSA group (*p* = 0.02). The incidence of glomerular sclerosis and interstitial fibrosis (ci) were higher in the Non-DSA group (*p* < 0.05). Other markers of acute (g, ptc, v) or chronic (ct, ah, cv) lesions were similar between groups (Table 2). Analysis of the biopsies performed within the first three months after transplantation showed no statistical difference between the groups in all other analyzed parameters. In biopsies performed after the 3^rd^ month, however, we observed a trend to increased frequency of glomerular sclerosis and interstitial inflammation in the Non-DSA group (*p* = 0.06), without difference in tubulitis (t) score. Other markers of acute (g, ptc, v) or chronic (ci, ct, ah, cv) lesions remained comparable between groups after three months post-transplant.

**Table 2:** Histological features of graft biopsies according to groups and the post-transplant time

	Total			0-3 months			> 3 months		
	Non- DSA	DSA+	*p*	Non- DSA	DSA+	*p*	Non- DSA	DSA+	*p*
Number of graft biopsies	38	29		20	19		18	10	
% glomerular sclerosis, median (min-max)	9.2 (0-58.3)	0 (0-17.6)	0.02	8.2 (0-54.5)	3.9 (0-15.4)	0.20	9.7 (0-58.3)	0 (0-17.6)	0.06
Banff scores (%)									
g 0	24 (63.1)	20 (68.9)	0.99	15 (75.0)	13 (68.4)	0.99	9 (50.0)	7 (70.0)	0.90
g 1-3	14 (36.9)	9 (31.1)		5 (25.0)	6 (31.6)		9 (50.0)	3 (30.0)	
i 0	21(55.3)	27 (93.1)	0.02	14 (70.0)	18 (94.7)	0.39	7 (38.9)	9 (90.0)	0.02
i 1-3	17 (44.7)	2 (6.9)		6 (30.0)	1 (5.3)		11 (61.1)	1 (10.0)	
t 0	27 (71.0)	27 (93.1)	0.27	19 (95.0)	19 (100.0)	0.91	8 (44.4)	8 (80.0)	0.50
t 1-3	11 (29.0)	2 (6.9)		1 (5.0)	0 (0.0)		10 (55.6)	2 (20.0)	
v 0	31 (81.6)	0 (0.0)	0.20	17 (85.0)	19 (100.0)	0.54	14 (77.8)	10 (100.0)	0.62
v 1-3	7 (18.4)	29 (100.0)		3 (15.0)	0 (0.0)		4 (22.2)	0 (0.0)	
ptc 0	27 (71.0)	24 (82.7)	0.87	15 (75.0)	16 (84.2)	0.97	12 (66.7)	8 (80.0)	0.96
ptc 1-3	11 (28.9)	5 (17.3)		5 (25.0)	3 (15.8)		6 (33.3)	2 (20.0)	
ct 0	13 (34.2)	12 (41.4)	0.98	8 (40.0)	8 (42.1)	0.99	5 (27.8)	4 (40.0)	0.98
ct 1-3	25 (65.8)	17 (58.6)		12 (60.0)	11 (57.9)		13 (72.2)	6 (60.0)	
ci 0	23 (60.5)	17 (58.6)	0.87	15 (75.0)	12 (63.1)	0.95	8 (44.4)	5 (50.0)	0.99
ci 1-3	15 (39.5)	12 (41.4)		5 (25.0)	7 (36.9)		10 (55.6)	5 (50.0)	
ah 0	33 (86.8)	18 (62.0)	0.23	19 (95.0)	12 (63.1)	0.19	14 (77.8)	6 (60.0)	0.91
ah 1-3	5 (13.2)	11 (38.0)		1 (5.0)	7 (36.9)		4 (22.2)	4 (40.0)	
C4d 0	31 (81.6)	17 (58.6)	0.37	16 (80.0)	12 (63.1)	0.85	15 (83.3)	5 (50.0)	0.47
C4d 1-3	7 (18.4)	12 (41.4)		4 (20.0)	7 (36.9)		3 (16.7)	5 (50.0)	
IF/TA 0	14 (36.8)	7 (24.1)	0.87	10 (50.0)	6 (31.6)	0.70	4 (22.2)	1 (10.0)	0.95
IF/TA 1-3	24 (63.2)	22 (75.9)		10 (50.0)	13 (68.4)		14 (77.8)	9 (90.0)	

min, minimum; max, maximum; g, glomerulitis; i, inflammation; t; tubulitis; v, intimal arteritis; ptc, peritubular capilatiris; ct, tubular atrophy; ci, interstitial fibrosis; ah, arteriolar hyalinosis; IF/TA, interstitial fibrosis and tubular atrophy; AMR, antibody mediated rejection; ACR, acute cell rejection.

The incidence of AMR was similar between groups, with one AMR case before the 3^rd^ month post-transplant in each group and one AMR case after the 3^rd^ month post-transplant in each group. However, we observed a higher incidence of acute cell mediated rejection in the Non-DSA group, mainly after 3 months post-transplant (Table 3).

**Table 3:** Biopsy-proven rejection, BK virus associated nephropathy, and calcineurin inhibitors nephrotoxicity according to groups and the post-transplant time

	Total			0-3 months			> 3 months		
	Non- DSA	DSA+	*p*	Non- DSA	DSA+	*p*	Non- DSA	DSA+	*p*
Number of graft biopsies	38	29		20	19		18	10	
AMR (%)	2 (5.2)	2 (6.9)	0.99	1 (5.0)	1 (5.2)	0.99	1 (5.55)	1 (10.0)	0.99
ACR (%)	12 (31.5)	2 (6.9)	0.02	3 (15.0)	1 (5.2)	0.60	9 (50.0)	1 (10.0)	0.04
Borderline	3	0		0	0		3	0	
1A	2	1		0	0		2	1	
1B	1	0		0	0		1	0	
2A	3	0		2	0		1	0	
2B	1	1		1	1		0	0	
3	2	0		0	0		2	0	
BKN (%)	3 (7.9)	0 (0.0)	0.66	0 (0.0)	0 (0.0)		3 (16.6)	0 (0.0)	0.76
CI nephrotoxicity (%)	1 (2.6)	1 (3.4)	0.25	0 (0.0)	0 (0.0)		1 (5.5)	1 (10.0)	0.99

AMR, antibody mediated rejection; ACR, acute cell rejection; BKN, BK virus associated nephropathy; CI, calcineurin inhibitors.

### DSA MFI levels and renal morphology

In our study, three biopsies were indicated by DSA (*de novo* or increase in preexisting antibody intensity). In Non-DSA group, one patient developed *de novo* DSA-DQ (> 10,000 MFI), without histological evidence of ACR or AMR. In the DSA+ group, one patient presented an increase in DSA-DQ fluorescence intensity (MFI from 5,400 to 6,200), without signs of rejection in graft biopsy. Another patient presented an increase in preformed DSA-DQ MFI (from 1,300 to 16,530), associated with *de novo* DSA-A and DSA-DR (sum of MFI 27,000) with histological evidence of AMR 2 and RCA 2B in graft biopsy.

### Clinical outcomes according to anti-HLA antibodies before transplantation

**Figure 1:**
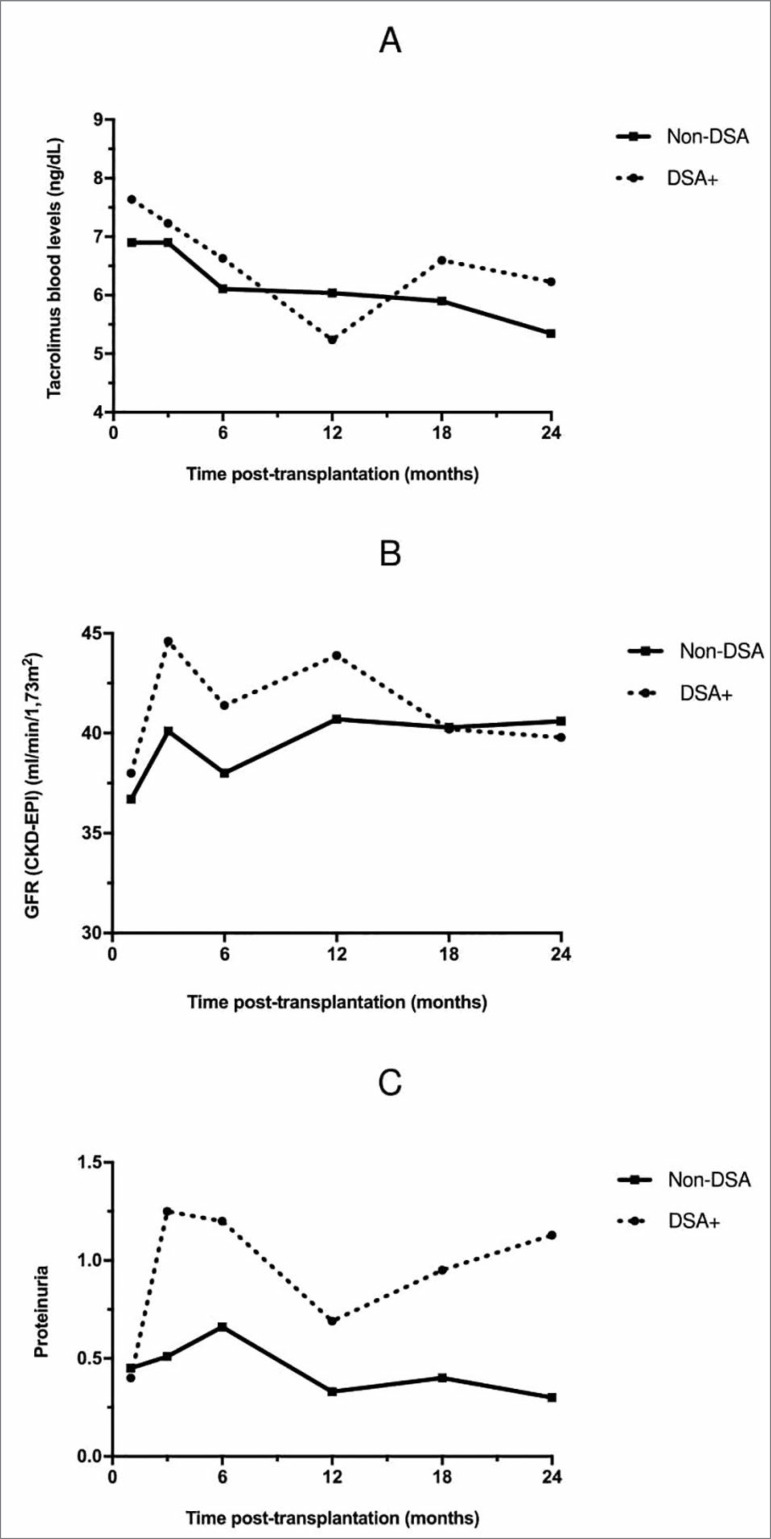
Tacrolimus blood level (A), glomerular filtration rate (GFR) (B), and proteinuria (C), according to the groups and the time posttransplantation.

Tacrolimus blood level was comparable between groups during follow up. (Figure 1A). There was no statistical difference in renal function between groups, in all time points (Figure 1B). The mean eGFR (CKD-EPI) in the first month post-transplant was 36.7 ± 21.9 mL/min/1.73m^2^ in the Non-DSA group, and 38.0 ± 20.8 mL/min/1.73m^2^ in the DSA+ group (*p* = 0.60). After 24 months of follow up, eGFR was 40.6 ± 13.8 mL/min/1.73m^2^ in the non-DSA group, and 39.8 ± 3.4 mL/min/1.73m^2^ in the DSA+ group (*p* = 0.46).

Although the DSA+ group presented higher levels of proteinuria than the Non-DSA group throughout the analyzed period, there was no significant difference between groups (Figure 1C).

## Discussion

In this study, the incidence of biopsy-proven AMR in patients with preformed anti-HLA antibodies, specific (DSA+) or non-specific (non DSA), was comparable. The analysis of Banff score of the groups showed statistically higher rates of interstitial inflammation (i) and fibrosis (ci) in the non-DSA group compared to the DSA+ group. Many contributing factors to interstitial fibrosis in kidney allograft have been cited in the literature, including donor characteristics, transplant parameters, calcineurin-inhibitor toxicity, hypertension, and rejection^10^. In our cohort, the characteristics of donors were similar between groups, as well as cold ischemia time, incidence of delayed graft function, and rejection in the first months after transplantation. After transplantation, the blood level of calcineurin inhibitor was also similar between groups, as well as the occurrence of biopsy-proven calcineurin inhibitor toxicity. Prednisone and mycophenolate doses also remained stable during follow-up. We observed a significantly higher incidence of acute cellular rejection in the Non-DSA group after the 3^rd^ month post-transplantation, and it may be associated with the histological findings in this period. In the Non-DSA group, three cases of BK-virus-associated nephropathy (BKAN) were diagnosed, with no cases in the DSA group. Interstitial inflammation occurred in over 70% of BKAN biopsies at diagnosis, correlating with viral histopathology and increasing early injury with accelerated fibrosis and tubular atrophy^11^. The higher incidence of polyomavirus infection could also explain the higher inflammation score observed in the Non-DSA group in our study.

Another possible contributing factor for the increased interstitial inflammation in the non-DSA group could be related to ischemia reperfusion injury (IRI). IRI is a set of histological changes resulting from tissue hypoxia and increased expression of inflammatory cytokines and adhesion molecules triggered by reperfusion, resulting in tubular cell apoptosis in reperfused tissue^12^. IRI is associated with an increased rate of acute rejection, DGF, primary non-function, and late graft dysfunction, leading to graft loss. Leukocytes participate in the development of the pathological processes involved in IRI, exacerbating tissue hypoxia by plugging capillaries and mediating direct cytotoxicity by producing oxygen radicals^12^. Dendritic cells and macrophages are key initiators, potentiators, and effectors of innate immunity in kidney IRI, inducing injury through inflammatory signals or directly through the release of soluble mediators^13^ Macrophages appear in the kidney within 1-5 days of IRI, and the early activation of macrophages and dendritic cells leads to the infiltration of neutrophils and generation of proinflammatory cytokines^13^

The only difference observed between groups was the doses of thymoglobulin, higher in the DSA + group. Although the optimal dosage of thymoglobulin used in the induction therapy is still not well established, previous studies showed that cumulative doses of thymoglobulin ranging from 4.2 to 7.4 mg/kg appear to be effective in prevention of acute rejection during the first year post-transplantation^14^. Nafar et al.^14^ compared three transplant recipient groups according to thymoglobulin dose: 4.5 mg/kg in 3 days, 4.5 mg/kg single bolus dose, and 6 mg/kg in 3 days. They found no significant difference in rejection among groups, but the incidence of glomerulitis and peritubular capilaritis was higher in the divided lower dose group. Increased doses of thymoglobulin can block different cell types, including B cells and dendritic cells, with a protective effect against IRI inflammatory injury, and this could be related to the findings of this study. Thus, the use of thymoglobulin could have an impact in reducing the incidence of IRI lesions, which also could contribute to the earlier differences between groups in inflammation and interstitial fibrosis in our study. Studies in animal models and in humans have shown that thymoglobulin increased the rate of apoptosis in white blood cells, protected the reperfusion tissue against IRI, and was beneficial in reducing DGF incidence and length of hospitalization^12^^,^ 15. Moreover, thymoglobulin causes energy and functional impairment of non-depleted lymphocytes and prevents migration of memory T-cells.

The most severe inflammation observed during the first months after transplantation tended to decrease during the first year. Most studies indicate that kidney morphology returns to almost normal after an ischemic insult. However, some degree of glomerular hypertrophy and interstitial scarring was observed in the long-term, progressing with glomerulosclerosis and IF/TA^16^. The higher intensity of glomerulosclerosis observed in the non-DSA group could be a consequence of the increased inflammatory response to IRI, with an imbalance between fibrosis and repair in tissue response. Although the characteristics of the donors were similar between the groups, it is not possible to rule out possible influence of donor histological changes in the graft glomerulosclerosis, since pre-transplant biopsies were not analyzed. Donor pre-implantation biopsies are routinely performed at the center, and kidneys are accepted only if glomerulosclerosis < 20% and with absence of arteriolar lesions. However, we observed a significant difference in glomerular sclerosis only after the 3^rd^ month post-transplantation, with no significant difference between groups in the first 3 months, which weakens the chance of donor lesions.

Another risk for poor outcome is the development of *de novo* DSA. However, its monitoring after transplantation is not well established^17^. The onset of new anti-HLA antibodies or the increase in the fluorescence intensity of existing antibodies could be related to the development of subclinical antibody-mediated rejections, and may lead to subtle histological changes in the absence of graft dysfunction or proteinuria. As protocol biopsies are not a routine in the center, the occurrence of subclinical rejection could be downsized.

Considering the biopsy findings according to Banff classification, both groups had minimal IF/TA (Banff IF/TA grade of 0 or 1) during the first two years after transplant. Gosset et al.^10^, studying 1,539 kidney transplant recipients, showed that patients with severe IF/TA (Banff grades 2 or 3) at 1 year post-transplantation were more likely to have circulating anti-HLA DSAs at the time of transplantation. These authors also demonstrated an association between the increase in DSA mean fluorescence intensity level (MFI) and augmented incidence of IF/TA, with severe IF/TA in presence of MFI > 5,000. In our study, only 3 biopsies were indicated by *de novo* DSA or increase in DSA MFI, but we were not able to find an association between MFI and IF/TA. Besides the low number of cases, this lack of association could be related to the capacity of the antibody binding to the complement, as C1q binding DSA are closely associated with acute AMR^4^. However, the lack of assessment of C1q status in our series impairs the correct evaluation of this hypothesis.

The clinical evolution in the first two years after transplantation was comparable between the two groups. Despite an increased incidence of acute cellular rejection in the Non-DSA group, lesions were mild, without negative impact in short term follow up. Only moderate to severe fibrosis is predictive of outcomes in most studies^2^, which could explain the evolution of renal function and proteinuria in our study, since the biopsy findings did not show significant fibrosis, according to IFTA score^8^

The lower incidence of grafts from deceased donors and the significantly lower incidence of expanded criteria donors and delayed graft function in the non-included group could be associated with better graft function and less proteinuria. This is an indication bias, since all biopsies were performed by clinical indication, such as impaired graft function or proteinuria, and it may have lessened the detection of subclinical rejections, limiting the conclusions of the study. Finally, the 2-year follow up period may have been insufficient to detect long-term changes in allograft tissue, renal function, and proteinuria. Despite these limitations, the present study revealed minimal interstitial fibrosis and tubular atrophy in early graft biopsy in recipients with preformed anti-HLA antibodies submitted to graft biopsy, which could be associated with immunosuppressive therapy. Prospective studies with protocol biopsies and post-transplant DSA monitoring in patients sensitized to the HLA system could elucidate the effect of the intensity and specificity of anti-HLA antibodies on tissue damage and development of graft fibrosis in this population.

## Conclusions

The incidence of antibody-mediated acute rejection in the first two years after transplantation was similar in a cohort of sensitized patients. The difference in inflammation and interstitial fibrosis scores between groups can be a consequence of an anti-inflammatory effect of higher doses of thymoglobulin in the DSA+ group, compared to an increased inflammation associated with acute cell-mediated rejection and BKAN in the Non-DSA group.
